# The Influence of Genetic and Environmental Factors among MDMA Users in Cognitive Performance

**DOI:** 10.1371/journal.pone.0027206

**Published:** 2011-11-16

**Authors:** Elisabet Cuyàs, Antonio Verdejo-García, Ana Beatriz Fagundo, Olha Khymenets, Joan Rodríguez, Aida Cuenca, Susana de Sola Llopis, Klaus Langohr, Jordi Peña-Casanova, Marta Torrens, Rocío Martín-Santos, Magí Farré, Rafael de la Torre

**Affiliations:** 1 Human Pharmacology and Clinical Neurosciences Research Group, Neurosciences Research Program, IMIM-Hospital del Mar Research Institute, Barcelona, Spain; 2 Universitat Autònoma de Barcelona (UAB), Barcelona, Spain; 3 Institute of Neuroscience, Universidad de Granada, Granada, Spain; 4 CIBER de Fisiopatología de la Obesidad y Nutrición (CB06/03), CIBEROBN, Hospital Clínico Universitario, Santiago de Compostela, Spain; 5 Epidemiology of Drugs of Abuse Research Group, Public Health and Epidemiology Research Program, IMIM-Hospital del Mar Research Institute, Barcelona, Spain; 6 Department of Statistics and Operations Research, Universitat Politècnica de Catalunya (UPC), Barcelona, Spain; 7 Behavioral Neurology and Dementias Research Group, Neurosciences Research Program, IMIM-Hospital del Mar Research Institute, Barcelona, Spain; 8 Disorders by Use of Substances Research Group, Neurosciences Research Program, IMIM-Hospital del Mar Research Institute, Barcelona, Spain; 9 Department of Psychiatry, Institute of Neurosciences, Hospital Clinic, IDIBAPS, CIBER-SAM, Barcelona, Spain; 10 Universitat Pompeu Fabra (CEXS-UPF), Barcelona, Spain; University of Bologna, Italy

## Abstract

This study is aimed to clarify the association between MDMA cumulative use and cognitive dysfunction, and the potential role of candidate genetic polymorphisms in explaining individual differences in the cognitive effects of MDMA. Gene polymorphisms related to reduced serotonin function, poor competency of executive control and memory consolidation systems, and high enzymatic activity linked to bioactivation of MDMA to neurotoxic metabolites may contribute to explain variations in the cognitive impact of MDMA across regular users of this drug. Sixty ecstasy polydrug users, 110 cannabis users and 93 non-drug users were assessed using cognitive measures of Verbal Memory (California Verbal Learning Test, CVLT), Visual Memory (Rey-Osterrieth Complex Figure Test, ROCFT), Semantic Fluency, and Perceptual Attention (Symbol Digit Modalities Test, SDMT). Participants were also genotyped for polymorphisms within the *5HTT*, *5HTR2A*, *COMT*, *CYP2D6*, *BDNF*, and *GRIN2B* genes using polymerase chain reaction and TaqMan polymerase assays. Lifetime cumulative MDMA use was significantly associated with poorer performance on visuospatial memory and perceptual attention. Heavy MDMA users (>100 tablets lifetime use) interacted with candidate gene polymorphisms in explaining individual differences in cognitive performance between MDMA users and controls. MDMA users carrying *COMT val/val* and *SERT s/s* had poorer performance than paired controls on visuospatial attention and memory, and MDMA users with *CYP2D6* ultra-rapid metabolizers performed worse than controls on semantic fluency. Both MDMA lifetime use and gene-related individual differences influence cognitive dysfunction in ecstasy users.

## Introduction

3,4-methylenedioxymethamphetamine (MDMA, ecstasy) is one of the most popular illegal psychostimulants abused among youth. There is compelling evidence that MDMA produces selective long-lasting serotonergic neuroadaptations, including regulatory changes in the expression of the serotonin transporter [1]–[3]. In humans, ligand-binding imaging studies have reported decreased serotonin transporter (SERT) binding throughout the cerebral cortices and the hippocampus in MDMA users compared to healthy controls [4], [5]. Furthermore, these studies have shown that decreased SERT binding is associated with lower memory performance in MDMA users. Although some studies have observed MDMA abstinence-related recovery of SERT availability in the midbrain and thalamus [6], [7] there is no data about SERT recovery in the cortex, and post-mortem evidence indicates that cortical SERT protein reductions can be more robust and durable than indicated by neuroimaging studies [8]. Overall these findings are suggestive of MDMA-induced neurotoxicity, which primarily affects the serotonin system and is linked to memory dysfunction.

Despite these findings about serotonin neuroadaptations, there is still debate on the question if MDMA use is reliably associated with neuropsychological impairment, regardless of the effects of concomitant use of other substances (e.g., cannabis, alcohol or other stimulants). Literature on this topic is characterized by considerable heterogeneity of results, which is attributable to the large amount of confounders inherent to research on the deleterious effects of MDMA [9]. Two meta-analyses of neuropsychological studies in MDMA users have concluded that MDMA use is robustly associated with learning and memory impairments [10], [11]. This conclusion is substantiated by evidence from studies showing dose-related detrimental effects of MDMA use on learning and memory [12]–[14]. Nonetheless, the size of these dose-related effects is modest (6–11% of explained variance [13], suggesting that other relevant individual differences, may play an important role in MDMA-induced neuropsychological deficits.

A number of genes affecting serotonin function, including *SERT* and *5HT2A* receptor gene polymorphisms, have demonstrated significant associations with cognition and may therefore importantly impact MDMA use related neuropsychological effects [15]. In addition, gene variants involved in MDMA pharmacodynamics and putative neurotoxic mechanisms, such as *COMT* [16], [17] and *CYP2D6* polymorphisms [18], and those impacting neural signaling pathways involved in learning and memory (e.g., *BDNF* and glutamate genes) [19]–[21] may also contribute to explain MDMA-induced neuropsychological deficits in humans [22]. Some of the dopamine and serotonin gene polymorphisms are equally relevant for MDMA-related cognitive effects based on their well-recognized role in modulating prefrontal cortex functioning and executive control [23], [24]. There is growing evidence that memory decrements in MDMA users are more neatly observable when neuropsychological probes involve a greater degree of complexity in terms of organization demands [25], [26]. These findings suggest that executive control processes linked to prefrontal systems may be impacted by the use of MDMA, and that genetic differences related to these systems may likely mediate these effects. In summary, different gene polymorphisms related to reduced serotonin function (*SERT s/s* and *5HT2a Tyr* genotypes), high enzymatic activity linked to the bioactivation of MDMA to neurotoxic metabolites (*COMT val/val* and CYP2D6 ultra-rapid metabolizers), and poor competency of executive control and memory consolidation systems (*COMT val/val*, *BDNF met/met*, and *GRIN2B C/C*) can contribute to explain variations in the cognitive impact of MDMA across regular users of this drug.

This study seeks to clarify the association between MDMA cumulative use and cognitive dysfunction, and the potential role of a number of relevant genetic polymorphisms in explaining individual differences in the cognitive effects of MDMA. To reach this aim, we examined cognitive performance in a sample of MDMA users recruited from a homogeneous socio-demographic context and thoroughly assessed to rule out psychiatric comorbidities. The sample also includes considerable variability in MDMA use patterns, which allowed us to characterize dose-related effects of cumulative MDMA use on cognitive performance. Neuropsychological testing was focused on those cognitive domains that have been consistently linked to MDMA use across studies: verbal and visual memory, attention/processing speed and executive functions. We hypothesize: (i) that heavier MDMA use would correlate with poorer neuropsychological performance in a dose-dependent fashion; (ii) that heavy MDMA users would perform poorer than cannabis and healthy comparison individuals on neuropsychological tests of processing speed, memory and fluency (indicating robust effects of MDMA on cognition regardless of co-abuse of cannabis); and (iii) that MDMA use would exacerbate cognitive performance decrements in individuals carrying genotypes associated with lower functionality of the serotonin, glutamate and dopamine systems.

## Methods and Materials

### Participants

Two hundred sixty-three Caucasian participants were recruited, of whom 60 were MDMA polydrug users, 110 were cannabis users and 93 were non-users. MDMA users were further classified into two subgroups according to their lifetime MDMA use applying a cut-off of 100 tablets (more than 100 tablets defined as heavy users) [27]. Volunteers were recruited through several sources: ‘word of mouth’, advertisement in the local university, and advertisement in the website of a local NGO (Energy Control) specialized in providing harm reduction guidelines among drug users. Potential participants were medically screened in the clinical research unit to rule out the presence of physical or neurological illness –as determined by standard physical examinations and biochemical determinations (supervised by the medical director –MFA). They were also carefully assessed to diagnose possible comorbid psychopathological disorders using a well-validated psychiatric interview –the Psychiatric Research Interview for Substance and Mental Disorders (PRISM; [28]) –which outcomes were supervised by two expert psychiatrists (MTM and RMS). Potential participants having medical illnesses or comorbid psychiatric disorders were excluded from the study. In addition, the PRISM interview also provides diagnoses for the whole spectrum of Diagnostic and Statistical Manual of Mental Disorders (DSM-IV) substance use disorders, such that we were able to rule out potential participants meeting criteria for abuse or dependence on drugs different than the ones targeted as the main aim of this study (MDMA and cannabis). Specifically, the following exclusion criteria were applied taking into account the data collected during the interview: for cannabis users, current history of regular use of other illegal drugs during last year, and past use of other illegal drugs for more than 5 occasions during lifetime; for non-users, current history of use of any illegal drugs during the past year, and past use of any illegal drugs in more than 5 occasions. Alcohol and nicotine use (but not abuse or dependence) was permitted. As for the MDMA group, because it was impossible to recruit exclusive MDMA users, we included MDMA users with exposure to other drugs if they did not meet abuse or dependence criteria for these other drugs.

### Test procedure

This study was approved by and conducted in accordance with the local ethics committee (CEIC-IMAS). Upon arrival to the research centre (IMIM, Hospital del Mar Research Institute), participants were informed of the ensuing protocol and provided informed consent before participating in the study.

All participants were subjected to an initial exploration that included a detailed medical history, biochemical analyses, physical examination, urine and hair toxicology screens, and a brief neurological examination. They were also assessed with a structured psychiatric interview specifically designed to diagnose lifetime use of different drugs and psychiatric comorbidity among substance users (PRISM [28]) administered by a psychiatrist or a clinical psychologist. Toxicology history in the past six months was confirmed by hair testing [29], [30]. All participants were requested to observe a 72 h abstinence period, and urine and hair drug screens were carried out by immunoassay (CEDIA, Thermo-Fisher) to confirm abstinence. Drug classes screened for included: cannabis, MDMA, cocaine and amphetamine/methamphetamine. A positive urine drug test excluded the participant for further assessments. This procedure allowed us to reliably classify participants into the different subgroups according to their pattern of drug use. All participants meeting inclusion criteria underwent a neuropsychological assessment session of 180 minutes, although here we only report analyses from a subset of these measures. All subjects were economically compensated for their participation.

### Cognitive measures

We administered the Vocabulary test from the WAIS-III (estimated IQ) [31], the California Verbal Learning Test (CVLT II) (verbal memory) [32], the Rey-Osterrieth Complex Figure Test (ROCFT) (visual spatial organization –Copy, and memory –Recall) [33], the Symbol Digit Modalities Test (attention/processing speed) [34], and the Category Word Fluency test [35]. A more detailed description of these tests can be found in [36].

### Genotyping

Genomic DNA was extracted from the peripheral blood leukocytes of all the participants using Flexi Gene DNA kit (Qiagen Iberia, S.L., Spain) according to the manufacturer's instructions.

*5HTTLPR* genotyping was performed using polymerase chain reaction (PCR) as described in [37]. The *5HTTLPR (A/G)* polymorphism (*rs25531*) was detected by MspI restriction enzyme digestion [38] with minor modifications. Briefly, 1 µl of the *5HTTLPR* PCR was digested in a 10 µl reaction assay containing 1× NEBuffer 2 and 3 U MspI at 37°C for 3 h and a final inactivation step of 20 minutes at 65°C. The resulting fragments were detected on an automatic ABI 3730XL capillary sequencer and analyzed by GeneMapper Software v3.5 (Applied Biosystems). Product sizes for the digest were: long A (L_A_) = 337 bp, short A (S_A_) = 292 bp, long G (L_G_) = 162 bp, and short G (S_g_) = 162 bp.

In some cases, were MspI digestion gave unclear results the samples were sequenced to assign the correct genotype. Sequencing was performed in both, the sense and antisense orientations. The excess primers and deoxynucleotides in the polymerase chain reaction (PCR) products were then degraded by adding a 2 µl of a solution of 0.8 units of shrimp alkaline phosphatase (New England Biolabs, Ipswich, MA), 4 units of *Escherichia coli* Exonuclease I (New England Biolabs) and 0.64× shrimp alkaline phosphatase buffer. The mixture was incubated at 37°C for 15 min, followed by deactivation for 15 min at 80°C. Sequencing reactions were performed with BigDye v3.1 (Applied Biosystems) in 10 µl total volume containing 1 µl template (approximately 25 ng), 3.2 pmol primer, 1 µl Applied Biosystems 5× DNA sequencing buffer, 2 µl BigDye v3.1, and water. The reactions were cycled at 94°C for 3 min, followed by 30 cycles at 96°C for 10 sec, 50°C for 5 sec, and 60°C for 4 min. Reactions were then purified with PureLink Quick Gel extraction kit (Invitrogen) according to manufacturer's instructions. Samples were analyzed on a Prism 3730xl DNA Analyzer (Applied Biosystems).

The *COMT val108/158met* (*rs4680*) and *BDNF val66met* (*rs6265*) single nucleotide polymorphism (SNP) allelic variants were determined using the 5′ exonuclease TaqMan assay with ABI 7900HT Sequence Detection System (Real Time PCR) supplied by Applied Biosystems. Primers and fluorescent probes were obtained from Applied Biosystems (TaqMan SNP Genotyping assays: assay ID C_2255335_10 and C_11592758_10 for rs4680 and rs6265, respectively). Reaction conditions were those described in the ABI PRISM 7900HT user's guide. Endpoint fluorescent signals were detected on the ABI 7900, and the data were analyzed using SDS software, version 2.3 (Applied Biosystems).

The *CYP2D6*, *GRIN2B C2664T* (*rs1806201*), the *5HT2A His452Tyr* (*rs6314*) and *T102C* (*rs6313*) genotypes were performed using the PHARMAchip™ DNA array (Progenika Biopharma, Derio, Spain) [39]. This DNA microarray allows the screening of genetic variants for phase I and phase II drug metabolism enzymes (DME), drug transporters, and drug protein effectors based on allele-specific oligonucleotide hybridization (ASO).

### Statistical Analyses

Sample characteristics, including drug consumption, are described by means of either mean and standard deviation (numerical variables) or absolute and relative frequencies (categorical variables). To quantify differences among the three groups, a generalization of Cohen's d, the standard mean difference for more than two groups, was computed. Following the suggestions of Cohen, values of 0.25 and 0.4 indicate medium and large effect sizes, respectively [40]. The chi-square test was applied to study the association between drug consumption (ecstasy consumption, cannabis consumption or control group) and each of the genotypes studied. In addition, it was used to check whether the Hardy-Weinberg equilibrium holds among each of the three populations under study. At a univariate level, the correlation between the cognitive performance and both lifetime ecstasy and lifetime cannabis consumption was quantified by Pearson's correlation coefficient among those individuals consuming ecstasy and those consuming cannabis, respectively. Lifetime consumption was measured in number of tablets (ecstasy) and number of joints (cannabis), respectively. These correlation coefficients were also computed for the subgroups defined by the *COMT val158met* genotype, on one hand, and the *5HTTLPR* genotype, on the other. Since several significant correlations with the neuropsychological variables were found (as described in the following section), in the sequel, a distinction was made between regular and heavy ecstasy users taking the consumption of 100 ecstasy pills during lifetime as cutoff. Since the principal interest was to study the association between cognitive performance and both drug consumption and each of the genotypes of interest, ANCOVA models were fitted for all neuropsychological variables and each genotype separately. These models included drug consumption group and the respective genotype as predictive variables of interest as well as gender and the WAIS-III Vocabulary index score; the Vocabulary index was used as a proxy of cultural level and verbal IQ. These two variables, sex and WAIS-III Vocabulary index, were included in all ANCOVA models in order to rule out the possible confusion due to differences observed among the drug consumption groups with respect to sex and education/IQ. Initially, all models did also include the two-way interaction between genotype and drug consumption. Whenever the interaction could be discarded, both factors were studied separately using the ANCOVA model excluding interaction. If a significant effect was observed of either factor, post-hoc multiple comparisons were carried out in the framework of the corresponding model using the Tukey test. If, by contrast, the interaction was significant, the effect of drug consumption was studied separately for each genotype expression and, vice versa. Again, the Tukey test for multiple comparisons was applied for these analyses in the framework of the ANCOVA models including interaction.

Statistical significance was set at 0.05 for the two-way genotype-drug consumption interaction within all ANCOVA models in order to reduce the probability of a possible Type II error. For those variables showing a significant interaction effect, results from follow-up pairwise comparisons were thresholded at p< = 0.01 to protect against Type I error was applied. The statistical software package R (The R Foundation for Statistical Computing), version 2.11.1, was used for all analyses. In particular, R Package multcomp [41] was used for the multiple pairwise comparisons.

## Results

### Sample characteristics

Socio-demographic variables, drug use characteristics and genotype distributions are presented in Table 1. Largest standardized mean differences (SMD) among the three samples were observed with respect to smoking habits (SMD = 0.69) and age of first alcohol use (SMD = 0.48). In addition, the groups differed notably in terms of the WAIS-III score (SMD = 0.3) and age (SMD = 0.25) with highest values among controls (average WAIS-III score of 12.6) and MDMA users (23.2 years), respectively. Despite the differences observed, neither age nor smoking or age of first alcohol consumption were included in the regression models. These characteristics were not considered possible confounders because of the relatively small interquartile range of age (20–24 years) and the lack of correlation of the other two variables with the neuropsychological variables under study (results not shown), respectively.

**Table 1:** Demographic variables, drug consumption characteristics.

	MDMA (n = 60)	Cannabis (n = 110)	Control (n = 93)	
	n (%)	n (%)	n (%)	SMD^c^
**Age** a	23.2 (3.2)	21.6 (2.7)	22.8 (4.1)	0.25
**Vocabulary WAIS-III** a	11.4 (2.4)	11.5 (2.1)	12.6 (2.0)	0.30
**Gender**				
Male	33 (55.0)	69 (68.2)	49 (52.7)	0.11
Female	27 (45.0)	41 (37.3)	44 (47.3)	
**University Degree** b				
Yes	41 (68.3)	75 (68.2)	83 (90.2)	0.30
No	19 (31.7)	35 (31.8)	9 (9.8)	
**Employment Status**				
Employed	17 (28.3)	29 (26.6)	26 (28.3)	0.04
Unemployed	13 (21.7)	24 (22.0)	13 (14.1)	
Student	30 (50.0)	56 (51.4)	53 (57.6)	
**Smoker**				
Current Smoker	46 (76.7)	70 (63.6)	17 (18.7)	0.69
Non smoker/Ex-smoker	14 (23.3)	40 (36.4)	74 (81.3)	
**Age at first tobacco use** a	16.4 (3.4)	18.5 (3.1)	18.1 (3.3)	0.34
**Cigarettes per day** a	11.1 (5.9)	8.9 (6.3)	6.8 (5.3)	0.36
**Age at first alcohol use** a	14.5 (1.8)	14.8 (1.4)	15.9 (1.4)	0.48
**Years of alcohol consumption** a	8.7 (3.0)	6.7 (2.8)	6.8 (4.4)	0.32
**Age at first cannabis use** a	15.6 (2.0)	15.5 (1.6)		0.02
**Years of cannabis consumption** a	7.7 (2.9)	6.1 (2.8)		0.56
**Age at first MDMA use** a	18.0 (2.9)			
**Years of MDMA consumption** a	5.2 (3.2)			

a Mean (SD).

b Including students.

c Standardized mean difference.

### Genotype distributions

Genotype distributions by group are presented in Table 2. The tests of Hardy-Weinberg equilibrium among the three different groups showed equilibrium for all genotypes except for the *COMT val158met* polymorphism in the cannabis group (*p*<0.01).

**Table 2:** Genotype distributions of the participants.

	MDMA heavy	MDMA light	MDMA	Cannabis	Control	*p*-value^a^
***5HTTLPR***						
L/L	7 (25.0)	9 (28.1)	16 (26.7)	31 (28.2)	29 (31.2)	0.121*0.280
L/S	12 (42.9)	12 (37.5)	24 (40.0)	58 (52.7)	49 (52.7)	
S/S	9 (32.1)	11 (34.4)	20 (33.3)	21 (19.1)	15 (16.1)	
***5HTTLPR+rs25531*** (n = 259)						
High (La/La)	5 (17.9)	9 (29.0)	14 (23.7)	25 (23.4)	21 (22.6)	0.478*0.605
Medium (La/Lg+La/S)	13 (46.4)	12 (38.7)	25 (42.4)	55 (51.4)	52 (55.9)	
Low (Lg/Lg+Lg/S+S/S)	10 (35.7)	10 (32.3)	20 (33.9)	27 (25.2)	20 (21.5)	
***rs25531*** (n = 259)						
A/A	-	-	53 (89.8)	93 (86.1)	77 (83.7)	0.568
G	-	-	6 (10.2)	15 (13.9)	15 (16.3)	
***5HT2A*** ** receptor his452tyr** (n = 259)						
His/His	21 (77.8)	21 (65.6)	42 (71.2)	59 (54.1)	59 (63.4)	0.598*0.529
His/Tyr	6 (22.2)	11 (34.4)	17 (28.8)	50 (45.9)	34 (36.6)	
***5HT2A*** ** receptor T102C**						
T/T	-	-	9 (15.3)	25 (22.9)	17 (18.7)	0.448
T/C	-	-	28 (47.5)	45 (41.3)	48 (52.7)	
C/C	-	-	22 (37.3)	39 (35.8)	26 (28.6)	
***BDNF*** ** val66met** (n = 262)						
val/val	22 (78.6)	20 (62.5)	42 (70.0)	59 (54.1)	59 (63.4)	0.109*0.109
met	6 (21.4)	12 (37.5)	18 (30.0)	50 (45.9)	34 (36.6)	
***GRIN2B*** ** C2664T** (n = 259)						
C/C	19 (70.4)	19 (59.4)	38 (64.4)	57 (52.3)	54 (59.3)	0.288*0.360
T	8 (29.6)	13 (40.6)	21 (35.6)	52 (47.7)	37 (40.7)	
***COMT*** ** val158met**						
val/val	12 (42.9)	9 (28.1)	21 (35.0)	26 (23.6)	27 (29.0)	0.069*0.037
val/met	13 (46.4)	13 (40.6)	26 (43.3)	70 (63.6)	45 (48.4)	
met/met	3 (10.7)	10 (31.2)	13 (21.7)	14 (12.7)	21 (22.6)	
**CYP2D6**						
Poor/Intermediate	-	-	9 (16.4)	14 (14.4)	13 (16.2)	0.928
Extensive/ultra-rapid	-	-	46 (83.6)	83 (85.6)	67 (83.8)	
**Genotype Combinations**						
***5HTTLPR*** **+** ***COMT*** ** val158met**						
L+met	-	-	24 (40.0)	68 (61.8)	57 (61.3)	0.075
L+val/val	-	-	16 (26.7)	21 (19.1)	21 (22.6)	
S/S+met	-	-	15 (25.0)	16 (14.5)	9 (9.7)	
S/S+val/val	-	-	5 (8.3)	5 (4.5)	6 (6.5)	
***5HTTLPR*** **+** ***BDNF*** ** val66met** (n = 262)						
L+met	-	-	13 (21.7)	41 (37.6)	27 (29.0)	**0.042**
L+val/val	-	-	27 (45.0)	47 (43.1)	51 (22.6)	
S/S+met	-	-	5 (8.3)	9 (8.3)	7 (7.5)	
S/S+val/val	-	-	15 (25.0)	12 (11.0)	8 (8.6)	
***5HTTLPR*** **+** ***5HT2A*** ** his452Tyr** (n = 259)						
L+His/His	-	-	27 (45.8)	70 (64.2)	56 (61.5)	0.070
L+Tyr	-	-	21 (20.3)	18 (16.5)	20 (22.0)	
S/S	-	-	20 (33.9)	21 (19.3)	15 (16.5)	

Results as the number of subjects (n) and percentage per genotype (%).

a The first of both values corresponds to the comparison of ecstasy users, cannabis users, and controls. The second (with an *) corresponds to the comparison of heavy ecstasy users, light ecstasy users, cannabis users, and controls.

No significant differences were observed in the genotype distributions among the different groups, except for the *5HTTLPR* and the *COMT val158met* polymorphisms. Significant differences were observed regarding the genotype distributions for the *COMT val158met* polymorphism (*p*<0.05) when the distinction between heavy and light MDMA users was taken into account. There were a higher number of individuals with the *val/val* genotype in the heavy MDMA users group (42.9%) compared to the control group (29.0%). The *val/met* genotype was also overrepresented in the cannabis group (63.6%) compared to the control group (48.4%) (*p*<0.05).

### *Correlations between neuropsychological variables and MDMA/cannabis lifetime consumption*

MDMA lifetime use showed a significant negative association with ROCFT Copy Accuracy (r = −0.604, IC95%: [−0.744, −0.413]), Immediate Recall (r = −0.391, IC95%: [−0.587, −0.152]), and Delayed Recall (r = −0.464, IC95%: [−0.642, −0.238]). MDMA lifetime use was also significantly correlated with SDMT (r = −0.269, IC95%: [−0.489, −0.016]). No other correlations reached statistical significance.

### Neuropsychological performance by group and genotype analyses

Results are presented in tables 3 and 4.

**Table 3:** Neuropsychological performance as a function of drug use status. Results are presented as mean (standard deviation).

Group	Ecstasy heavy (n = 28)	Ecstasy light (n = 32)	Cannabis (n = 110)	Control (n = 93)	*p*-value
**CVLT**					
Immediate Recall	11.3 (2.5)	11.6 (2.6)	11.6 (2.5)	12.5 (2.1)	0.301
Delayed Recall	11.5 (2.3)	11.7 (2.4)	11.6 (2.6)	12.6 (2.2)	0.221
Total Recognition	14.5 (0.9)	14.2 (0.9)	14.1 (1.2)	14.4 (1.0)	0.279
Total A1–A5	50.9 (8.3)	53.5 (8.6)	52.8 (8.3)	55.8 (6.7)	0.211
**ROCFT**					
Copy Accuracy	31.6 (3.3)	34.1 (1.9)	34.5 (2.5)	34.5 (2.2)	**0.000**
Immediate Recall	20.5 (5.6)	24.7 (4.5)	24.9 (5.2)	25.2 (4.9)	**0.001**
Delayed Recall	20.6 (5.5)	24.4 (4.4)	24.9 (5.0)	24.7 (4.9)	**0.002**
**SDMT**	55.8 (10.1)	59.2 (11.9)	59.7 (9.2)	61.6 (10.4)	0.185
**Semantic Fluency**	23.6 (3.5)	23.8 (5.4)	24.7 (5.6)	26.3 (6.3)	0.218

**Table 4:** Neuropsychological performance as a function of gene x drug use interaction.

	Groups				
	Ecstasy heavy (n = 28)	Ecstasy light (n = 32)	Cannabis (n = 110)	Control (n = 93)	*p*-value
**ROCFT – copy accuracy**					
*COMT val158me*t					
*val*/*val*	32.2 (3.4)	33.2 (1.8)	33.5 (3.8)	35.1 (1.5)	**0.020**
*val*/*met*	31.1 (3.5)	34.5 (1.9)	34.7 (1.9)	34.7 (1.6)	
*met*/*met*	31.7 (2.1)	34.3 (1.9)	35.1 (1.8)	33.2 (3.2)	
*COMT val158met* and *5HTTLPR* genotypes combinations					
*L*+*met*	32.8 (2.8)*		34.8 (1.9)	34.2 (2.4)	**0.014**
*L*+*val*/*val*	33.2 (2.3)*		33.0 (4.1)	34.9 (1.7)	
*S*/*S*+*met*	33.6 (3.2)*		34.9(1.7)	34.6 (1.4)	
*S*/*S*+*val*/*val*	30.8 (3.7)*		35.6 (0.9)	35.8 (0.4)	
**ROCFT – immediate recall**					
*COMT val158met* and *5HTTLPR* genotypes combinations					
*L*+*met*	23.0 (5.4)*		24.2 (5.3)	24.6 (5.2)	**0.015**
*L*+*val*/*val*	22.6 (5.4)*		26.5 (5.0)	26.9 (4.2)	
*S*/*S*+*met*	24.2 (5.2)*		26.4 (4.3)	22.2 (3.7)	
*S*/*S*+*val*/*val*	17.5 (4.1)*		23.6 (5.0)	29.4 (1.6)	
**ROCFT – delayed recall**					
*COMT val158met* and *5HTTLPR* genotypes combinations					
*L*+*met*	22.6 (5.3)*		24.2 (5.3)	24.3 (5.1)	**0.011**
*L*+*val*/*val*	22.6 (5.4)*		25.8 (4.8)	26.4 (4.1)	
*S*/*S*+*met*	24.5 (4. 9)*		26.6 (4.1)	21.1 (3.5)	
*S*/*S*+*val*/*val*	18.0 (3.8)*		24.9 (4.2)	28.6 (2.5)	
**SDMT**					
*5HTTLPR*					
*L*/*L*	56.7 (9.2)	57.2 (7.5)	57.1 (9.5)	62.1 (11.1)	**0.016**
*L*/*S*	59.2 (10.6)	62.8 (14.1)	60.0 (8.4)	59.7 (9.1)	
*S*/*S*	50.4 (8.6)	56.7 (12.1)	62.6 (10.2)	66.9 (11.9)	
*COMT val158met* and *5HTTLPR* genotypes combinations					
*L*+*met*	58.5 (10.6)*		58.4 (9.0)	60.3 (10.7)	**0.001**
*L*+*val*/*val*	60.9 (11.5)*		61.0 (8.2)	61.5 (7.3)	
*S*/*S*+*met*	57.5 (9.6)*		63.9 (10.1)	62.7 (6.0)	
*S*/*S*+*val*/*val*	43.2 (7.1)*		58.4 (10.8)	73.2 (16.0)	
**Semantic Fluency**					
CYP2D6 Phenotype					
Intermediate/Poor	21.7 (4.7)*		25.7 (6.0)	22.5 (6.6)	**0.047**
Ultra-rapid/Extensive	24.0 (4.7)*		25.0 (5.6)	27.5 (5.9)	

*Refers to the MDMA group irrespective of the lifetime consumption (n = 60).

Results are presented as mean (standard deviation).

### Verbal Memory (CVLT)

We found no effects of group, genotype or the group x genotype interaction on performance indices from this test.

### Visual Memory (ROFCT)

#### Copy Accuracy

We found a main group effect; paired contrasts indicated that heavy MDMA users had lower scores than light MDMA users (t = 4.1, df = 58, *p*<0.001), cannabis users (t = 5.6, df = 136, *p*<0.0001), and non-users (t = 5.7, df = 119, *p*<0.0001).

We observed a significant group x genotype interaction for the *COMT val158met* (*p*<0.05). When the *val/met* genotype was examined, heavy MDMA users with this genotype had lower scores than light MDMA users (t = 4.7, df = 58, *p*<0.0001), cannabis users (t = 5.9, df = 136, *p*<0.0001) or non-users (t = 6.1, df = 119, *p*<0.0001) with the same genotype.

When considering the combination of the *COMT val158met* and *5HTTLPR* genotypes, we also found an effect of the group x genotype interaction (F = 2.7, df1 = 6, df2 = 249, *p* = 0.014). MDMA users carrying the *S/S+val/val* genotype scored poorer than non-users carrying the same genotype (t = 3.5, df = 9, *p* = 0.01). In addition, concerning the *L+met* genotype, MDMA users scored poorer than Cannabis users (t = 3.3, df = 90, *p* = 0.001).

### Immediate Recall

We found a main effect of group, with heavy MDMA users having significantly lower scores than light MDMA users (t = 3.2, df = 60, *p*<0.01), cannabis users (t = 4, df = 136, *p*<0.001), and non-users (t = 3.7, df = 119, *p*<0.01).

When examining the combination of the *COMT* val158met and *5HTTLPR* genotypes, we found a group x genotype effect (F = 2.7, df1 = 6, DF2 = 249, *p* = 0.015); MDMA individuals with the *S/S+val/val* genotype had significantly lower scores than non-users carrying the same genotype (t = 4.5, df = 9, *p*<0.01).

#### Delayed recall

We found a main group effect (*p*<0.01), with heavy MDMA users having significantly lower scores than cannabis users (t = 3.9, df = 136, *p*<0.001), and non-users (t = 3.2, df = 119, *p*<0.01). We also found a main effect of *5HT2A* genotype, indicating that individuals carrying the *His/Tyr* variant had significantly poorer performance than those with the *His/His* genotype (t = 2.4, df = 252, *p* = 0.01).

The study of the interaction between the *COMT val158met* and *5HTTLPR* genotypes showed an effect of the group x genotype interaction (*p* = 0.01). Pairwise comparisons showed that MDMA users carrying the *S/S+val/val* combination had significantly lower scores than non-users (t = 4.2, df = 9, *p*<0.01).

### Attention/Speed (SDMT)

We found no significant main effect of group on performance in this test.

We found a significant group x *5HTTLPR* genotype interaction (F = 2.7, df1 = 6, df2 = 249, *p* = 0.016), with heavy MDMA users carrying the *S/S* genotype performing poorer than *S/S* non-users (t = 3.1, df = 33, *p* = 0.01).

In addition, we observed a significant group x genotype interaction for the combination of *5HTTLPR* and the *COMT val158met* genotypes (F = 4.1, df1 = 6, df2 = 249, *p* = 0.001). Pairwise comparisons showed that MDMA users carrying the *S/S+val/val* combination performed significantly more poorly than MDMA *L+val/val* carriers (t = 3.2, df = 19, *p*<0.01).

### Semantic Fluency

The semantic word fluency was unaffected by group or genotypes alone. However, there was a significant effect of the group x *CYP2D6* phenotype interaction (F = 3.1, df1 = 2, df2 = 224, *p*<0.05). In non-users, individuals who were Intermediate/Poor for the *CYP2D6* performed worse than those who were Ultra-rapid/Extensive (t = 2.8, df = 78, *p*<0.01). In contrast, MDMA users with the Ultra-rapid/Extensive phenotype had significantly lower scores than those with the same phenotype in the non-user group (t = 2.8, df = 111, *p*<0.01).

## Discussion

Our findings show detrimental effects of both MDMA lifetime use and variations in candidate genes on a number of neuropsychological measures, with particular relevance of visuospatial attention and memory. With respect to dose-related effects, we found that greater lifetime use of MDMA is negatively correlated with performance on visuospatial memory (ROCFT) and attention and perceptual speed (SDMT) tests. These results were further supported by group comparisons, which showed that heavy MDMA users (lifetime use>100 tablets) have significantly poorer visuospatial memory than light MDMA users, cannabis users and non-users. Importantly, we found a number of gene x MDMA interaction effects. Results for *COMT* and *SERT* genes showed that MDMA users carrying the *COMT val/val* and *SERT S/S* genotypes have poorer performance on tests of visuospatial memory and perceptual attention (ROCFT and SDMT). Delayed recall was also modulated by a gene polymorphism in the *5HT2a* receptor, such that *Tyr* carriers had poorer performance irrespective of drug use. Finally, we found an interaction between MDMA use and CYP2D6 extra-high metabolic activity phenotype and lower performance on verbal fluency.

The main MDMA dose-related findings and MDMA x gene interactions were found in the ROCFT. This is a complex task involving visuospatial attention and planning/organization skills during the copy, and planning and episodic memory skills during immediate and delayed recall [42]. Copy performance is associated with dorsolateral prefrontal cortex (DLPFC) and parietal cortex functioning [43], whereas immediate and delayed recall are associated with the functioning of the DLPFC [44] and the hippocampus [45]. Therefore, our findings point to a substantial impact of MDMA lifetime amount of use on fronto-parietal and fronto-temporal systems supporting attentional and memory functions, in agreement with previous neuropsychological and neuroimaging findings [46]–[48]. The fact that the larger dose-related correlations are found for the Copy index indicates that MDMA cumulative use may have greater detrimental effects on visuospatial attention and organization skills than on recall *per se*; this is consistent with the finding that MDMA use is also negatively associated with SDMT-indexed perceptual attention, and with decreased gray matter volumes and SERT availability in posterior brain cortices [48].

The association between MDMA use and perceptual attention and visual memory/organization skills seems to be modulated by *COMT* and *SERT* genotypes. Our results consistently showed that MDMA users carrying the *COMT val/val* and *SERT S/S* genotypes perform significantly more poorly on ROCFT Copy, Immediate and Delayed Recall, and on SDMT, such that there was consistency between gene x drug effects on different probes involving perceptual attention and visuospatial planning/memory skills. These results fit with evidence showing that the *COMT* gene is significantly associated with visuospatial planning ability –gene-carriers with high enzymatic activity display poorer performance [49] and reduced DLPFC and parietal activation during planning tasks [50]. They are also in agreement with neuropsychological outcomes in individuals with microdeletion of chromosome 22q11.2 –in which the *COMT* is one of the genes in the deleted region– displaying robust deficits in the ROCFT [43]. There is also evidence of the influence of the *SERT* genotype on visuospatial attention/planning performance indexed by a Mental Rotation task after tryptophan depletion –which mimics MDMA-induced serotonin reductions [51]. Furthermore, MDMA chronic use is robustly associated with loss of *SERT* availability in occipital, hippocampal and parietal regions [48], all networks involved in perceptual/attentional processing and memory. Overall, our results are suggestive of the notion that heavy MDMA use and *COMT val/val* and *SERT S/S* genotypes interact to produce greater detrimental effects on perceptual attention and planning/organization, ultimately affecting visuospatial memory skills. However, these results would need follow-up in larger samples, and they need to be interpreted with caution considering that the role of the *COMT* gene on cognition and brain functioning is still controversial [52], [53]. In the case of delayed recall, performance was further modulated by a *5HT2a* gene polymorphism, such that *Tyr* carriers have poorer performance irrespective of drug use; this is in agreement with previous observations on the association between this genotype and the consolidation process of episodic memory [54].

In agreement with our initial assumptions, MDMA users with CYP2D6 high metabolic activity phenotypes performed more poorly on the semantic fluency test. These results are in agreement with recent findings about the link between higher CYP2D6 activity and impaired executive performance, including semantic fluency, in methamphetamine users [55]. In this case, we extend their findings by showing specific effects of the Ultra-rapid/Extensive phenotype on executive performance in MDMA users, a drug-using group in which greater cognitive dysfunction was expected based on specific pharmacodynamic mechanisms [56]. Furthermore, fluency is one of the executive skills more consistently impaired in MDMA users [57], conferring clinical significance to this gene x drug interaction effect. This finding supports the proposal that *CYP2D6* polymorphisms may modulate MDMA-induced neurotoxic effects [17] and subsequent decrements in executive performance. More research including additional executive phenotypes and larger sample sizes are warranted to further substantiate these promising findings. Three other polymorphisms –the *BDNF val/met*, the *5HT2a T102C*, and the *GRIN2B C2664T*– were explored but failed to show significant results in relation to cognitive performance. Nonetheless, the *GRIN2B* genotype showed a trend to significance in relation to verbal memory (*T allele* carriers recalling more words), raising the possibility that effects of these genes on cognition may emerge in larger samples sizes.

In conclusion, this study reliably demonstrates dose-related effects of MDMA use on visual attention, organization and memory. In addition, we show an interaction between MDMA use and different gene polymorphisms in determining poorer performance of MDMA users in tests of visual attention and memory (*COMT* and *SERT* genes) and verbal fluency (*CYP2D6*). Strengths from the study include the successful recruitment of a large number of non-treatment seeking MDMA users who self-report an adequate academic/ professional and social functioning, thus avoiding socio-demographic confounders and approximating research to the reality of MDMA use beyond clinical settings; the carefully conducted medical and psychiatric explorations to discard potential confounders related to physical illness or psychopathology; and the use of well-validated neuropsychological measures taxing key cognitive domains related to MDMA use. Limitations include the relatively small sample size for a genetic study –especially when combining some of the rare genotypes, and the elevated number of statistical comparisons, which may have inflated the risk of Type I error. Nonetheless, we should note that our drugs x gene interaction findings are in agreement with initial assumptions, biologically plausible and overly consistent with previous literature, and consistent across independent but conceptually related neuropsychological probes and indices. Therefore, although these results would need to be further explored and replicated, we arguably reckon that the probability that they stem from false positive effects is low.
